# Silencing of AURKA augments the antitumor efficacy of the AURKA inhibitor MLN8237 on neuroblastoma cells

**DOI:** 10.1186/s12935-019-1072-y

**Published:** 2020-01-07

**Authors:** Yan Yang, Lili Ding, Qi Zhou, Li Fen, Yuhua Cao, Junjie Sun, Xuefeng Zhou, Aiguo Liu

**Affiliations:** 1 Experimental Medicine Center of Tongji Hospital, Tongji Medical College, Huazhong University of Science & Technology, Wuhan, 430030 China; 2 Department of Pediatrics of Tongji Hospital, Tongji Medical College, Huazhong University of Science & Technology, Wuhan, 430030 China; 3 Department of Pediatric Surgery of Tongji Hospital, Tongji Medical College, Huazhong University of Science & Technology, Wuhan, 430030 China

**Keywords:** MLN8237, Neuroblastoma, Aurora kinase A, Small interfering RNA, Cellular senescence

## Abstract

**Background:**

Aurora kinase A (AURKA) has been implicated in the regulation of cell cycle progression, mitosis and a key number of oncogenic signaling pathways in various malignancies including neuroblastoma. Small molecule inhibitors of AURKA have shown potential, but still not as good as expected effects in clinical trials. Little is known about this underlying mechanism. Here, we evaluated the inhibitory effects of AURKA inhibitor MLN8237 on neuroblastoma cells to understand the potential mechanisms responsible for tumor therapy.

**Methods:**

MLN8237 treatment on neuroblastoma cell line IMR32 was done and in vivo inhibitory effects were investigated using tumor xenograft model. Cellular senescence was evaluated by senescence-associated β-gal Staining assay. Flow cytometry was used to tested cell cycle arrest and cell apoptosis. Senescence-associated signal pathways were detected by western blot. CD133 microbeads and microsphere formation were used to separate and enrich CD133^+^ cells. AURKA small interfering RNA transfection was carried to downregulate AURKA level. Finally, the combination of MLN8237 treatment with AURKA small interfering RNA transfection were adopted to evaluate the inhibitory effect on neuroblastoma cells.

**Results:**

We demonstrate that MLN8237, an inhibitor of AURKA, induces the neuroblastoma cell line IMR32 into cellular senescence and G2/M cell phase arrest. Inactivation of AURKA results in MYCN destabilization and inhibits cell growth in vitro and in a mouse model. Although MLN8237 inhibits AURKA kinase activity, it has almost no inhibitory effect on the AURKA protein level. By contrast, MLN8237 treatment leads to abnormal high expression of AURKA in vitro and in vivo. Knockdown of AURKA reduces cell survival. The combination of MLN8237 with AURKA small interfering RNA results in more profound inhibitory effects on neuroblastoma cell growth. Moreover, MLN8237 treatment followed by AURKA siRNA forces senescent cells into apoptosis via suppression of the Akt/Stat3 pathway.

**Conclusions:**

The effect of AURKA-targeted inhibition of tumor growth plays roles in both the inactivation of AURKA activity and the decrease in the AURKA protein expression level.

## Background

Neuroblastoma (NB) is one of the most frequently occurring solid tumors in early childhood. The overall incidence is approximately one case in 7000 live births, and the median age at diagnosis is approximately 18 months. In addition, NB is responsible for approximately 13% of all pediatric cancer mortalities [1]. *MYCN*, an *MYC* family proto-oncogene, is amplified in 25% of neuroblastomas. Amplification of the *MYCN* marks high-risk disease. High-risk patients have a poor prognosis and need intense chemotherapeutic regimens. Despite the aggressive treatment, 50–60% of these patients will not achieve long-term cure owing to disease progression and resistance to current therapies [2]. Currently, as an undruggable target, there is no specific compound targeting MYC protein [3].

Aurora kinase A (AURKA) belongs to the mitotic serine/threonine kinase family, which is evolutionally conserved and is localized at the centrosome. AURKA is essential for many biological processes, including centrosome maturation and separation, spindle assembly, chromosome alignment and the G2 to M transition [4, 5]. It has been shown that AURKA is widely overexpressed in various tumors, including neuroblastoma (NB), and has been linked to a poor prognosis [6]. Furthermore, overexpression of AURKA is also closely associated with the overexpression of MYCN in NB. Studies have shown that AURKA can form a complex with MYCN to stabilize the MYCN structure and avoid its degradation, while inhibiting AURKA activity can promote the degradation of MYCN [7]. Therefore, targeting AURKA therapeutics can not only improve the effect of treating NB by inhibiting the activity of AURKA but also achieve the purpose of decreasing the MYCN protein. MLN8237, also known as alisertib, is an orally administered selective AURKA inhibitor that has shown potential anticancer effects in preclinical studies [8]. However, clinical trials cannot prove that MLN8237 is more effective than traditional chemotherapy drugs [9]. However, as a targeting drug, MLN8237 has a fewer side effects than common therapeutic drugs. Thus, despite disappointing early results, MLN8237 remains under investigation in a several cancer types both as monotherapy and in combination with traditional cytotoxic chemotherapy, with encouraging results [10].

Herein, we investigated the therapeutic effect of the AURKA inhibitor MLN8237 on neuroblastoma cells in vitro and in vivo. We observed that MLN8237 blocked the cell cycle at the G2/M phase and induced cell senescence. Senescent tumor cells stopped dividing, and tumor progression was controlled. We found that MLN8237 indeed inhibited AURKA activity, but it showed no inhibitory effect on the AURKA protein level. By contrast, MLN8237 treatment leads to abnormal high expression of AURKA in several neuroblastoma cell lines. Knockdown of AURKA using RNAi forced cells into apoptosis. The combination of MLN8237 with AURKA siRNA resulted in a more profound inhibitory effect on neuroblastoma cell growth in a mouse model. Knockdown of AURKA in the presence of MLN8237 pretreatment induced senescent cells into apoptosis by suppressing Akt/Stat3 activities. These results suggest that, to improve the effect of AURKA-targeted inhibition on neuroblastoma growth needs not only inactivation of AURKA but also downregulation of the AURKA protein level.

## Methods

### Cell culture and AURKA inhibitor

The human neuroblastoma cell lines IMR32, SK-N-BE, LAN-1, SK-N-SH and hepatocarcinoma cell line HepG2, and glioma cell line U373 were obtained from American Type Culture Collection (ATCC). All cell lines were cultured in DMEM medium supplemented with 10% fetal bovine serum and the antibiotics penicillin and streptomycin. The Aurora A kinase inhibitor MLN8237 (Alisertib, HY-10971) was purchased from Medchem Express (MCE). All other reagents were commercially available.

### Senescence-associated SA-β-gal staining assay

IMR32 cells were treated with 2 μmol/l of MLN8237, DMSO or no treatment as the control. At day 3, cellular senescence was evaluated using the senescence-associated SA-β-gal Staining Kit (GMS10012.1; GENEMED Scientifics) according to the protocol. Briefly, cells were washed twice with Reagent A and were fixed for 5 min with Reagent B at room temperature. Next, the cells were washed twice with Reagent C and were incubated at 37 °C for 3 to 16 h with the staining solution (Reagent D:Reagent E 19:1) before being observed using a microscope (Olympus).

### Cell cycle analysis

IMR32 cells were treated with 2 μmol/L of MLN8237 for 2 days. Afterwards, the cells were harvested and fixed with 70% ice-cold ethanol at − 20 °C for at least overnight. Next, the cell pellets were collected by centrifugation and were resuspended in PBS containing 100 µg/mL of RNaseA, 50 µg/mL of PI and 0.2% Triton X-100. After PI staining, quantification of the cell cycle distribution was carried out using a FACSCalibur flow cytometer equipped with FlowJo software.

### Cell apoptosis analysis

Cell apoptosis was detected using the Alexa Fluor^®^ 488 annexin V/Dead Cell Apoptosis Kit (Invitrogen). Briefly, after treatment with MLN8237, the cells were washed with cold PBS, were resuspended with 1 × binding buffer and then were incubated with Alexa Fluor^®^ 488 annexinV and PI for 15 min at room temperature. Next, cell apoptosis was analyzed by measuring the fluorescence emission at 530 nm and 575 nm using 488-nm excitation.

### MTT assay

In total, 5 × 10^3^ IMR32 cells were seeded in a 96-well plate. The next day, the cells were treated with MLN8237 and/or AURKA siRNA. Cell viability was tested by the MTT assay on day 1, day 2, day 3, day 4, day 5, and day 6 after treatment. Briefly, MTT solution was added to each well, and the cells were incubated at 37 °C for 4 h. The absorbance was finally determined at 490 nm using a microplate reader (BioTek, Vermont, USA). The cell viability was assessed and expressed as the relatively cell viability with the OD values. The experiment was repeated at least three times.

### Transfection and real-time RT-PCR

AURKA siRNAs were synthesized by RIBOBIO, and their sequences were as follows: AURKA siRNA-1: ATGCCCTGTCTTACTGTCA; AURKA siRNA-2: ATTCTTCCCAGCGCGTTCC. siN05815122147 NControl_05815 (standard) from RIBOBIO served as the siRNA control. For siRNA transfection, the cells were seeded into six-well plates at 2.0 × 10^5^ cells per well. On the following day, AURKA siRNA or siRNA control was transfected into cells using Lipofer3000 (Life Technologies) following the manufacturer’s instructions. For MYCN plasmid transfection, the MYCN overexpression plasmid was purchased from Vigene Biosciences and used following the manufacturer’s instructions. Plasmid transfection was conducted using Lipofer3000 reagent (Life Technologies). Total RNA was extracted from cells using Trizol reagent (Ambion; 15596026). Quantitative RT-PCR was executed using the One Step SYBR^®^ PrimeScript™ RT-PCR Kit II (Takara, RR086A) according to the manufacturer’s protocol. RT-PCR was performed using the Roche LightCycler 480II (Roche). The gene-specific primers are listed in Table 1. All PCRs were undertaken at least three times to ensure consistency.

**Table 1:** Primer sequence for real time RT-PCR

Primer name	Primer sequence (5′–3′)
P21 forward	CTTCGACTTTGTCACCGAGA
P21 reverse	GGTCCACATGGTCTTCCTCT
Bmi1 forward	CGTGTATTGTTCGTTACCTGGA
Bmi1 reverse	TTCAGTAGTGGTCTGGTCTTGT
CIAP2 forward	AAGCTACCTCTCAGCCTACTTT
CIAP2 reverse	CCACTGTTTTCTGTACCCGGA
NEFL forward	CGACAGCTTGATGGACGAAAT
NEFL reverse	GATCTGCGCGTACTGGATCTG

### Western blot analysis

Total cellular proteins were lysed using RIPA buffer supplemented with proteinase inhibitor cocktail and phosphatase inhibitor cocktail on ice for 20 min, followed by centrifugation at 12,000 rpm for 10 min at 4 °C. Next, the supernatants were collected. Equal amounts of proteins were subjected to SDS-PAGE and were transferred to nitrocellulose membranes. Next, the membrane was blocked with 5% milk in 0.1% TBST for at least 1 h at RT. Thereafter, the blots were incubated with primary antibody at 4 °C overnight. The antibodies used were as follows: anti-AURKA (abcam; ab52973; 1:30,000), anti-pAURKA(pT288) (abcam; ab52973; 1:1000), anti-MYCN (Novus Biologicals; NB200-109; 1:800), anti-pMYCN (S62) (abcam; ab185656; 1:1000), anti-MYCN (abcam; ab185655; 1:1000), anti-cyclinB (Arigo; ARG55257; 1:1000), anti-GSK3β(abcam; ab3239; 1:1000), anti-pGSK3β(Y216) (abcam; ab75745; 1:1000), anti-PTEN (abcam; ab32199; 1:10,000), anti-P27 (abcam; ab32034; 1:1000), anti-P53 (Genetex; GTX102965; 1:1000), anti-P21 (abcam; ab80633; 1:1000), anti-AKT (Arigo; A54929; 1:1000), anti-AKT(Ser473) (CST; #9271; 1:1000), anti-PI3K (abcam; ab86714; 1:1000), anti-RB (Arigo; ARG51103; 1:1000), anti-pRB (Ser795) (Arigo; ARG51631; 1:1000), anti-pRB(Ser807) (Arigo; ARG51632; 1:1000), anti-P16 (Genetex; GTX129903; 1:1000), anti-STAT3 (Genetex; GTX104616; 1:1000), anti-pSTAT3 (Tyr705) (Arigo; ARG51549; 1:1000), anti-JAK2 (Genetex; GTX101132; 1:1000), anti-pJAK2 (Y1007 + Y1008) (abcam; ab32101; 1:5000), anti-Bmi1 (Arigo; ARG55885; 1:1000), anti-survivin (Proteintech; 10508-1-AP; 1:1000), anti-GAPDH (abcam; ab8245; 1:10,000). Subsequently, the membranes were incubated with an HRP-conjugated secondary antibody (Cell Signaling Technology) at room temperature for 1 h and were visualized using enhanced chemiluminescence reagents (Sigma) according to the manufacturer’s instructions.

### Tumor growth in xenografts

In vivo experiments were carried out according to protocols approved by the Ethical Committee of Huazhong University of Science and Technology, People’s Republic of China. All animals were housed under 12-h light/dark conditions with free access to food and water according to the criteria outlined in the “Guide for the Care and Use of Laboratory Animals” prepared by the National Academy of Sciences and published by the National Institute of Health (NIH publication 86–23, revised 1985). Female athymic nude mice 6–8 weeks of age (SJA Lab Animal, Hunan, China) were used for the tumor xenograft model. In total, 5 × 10^6^ IMR32 cells were subcutaneously inoculated in the right flank of each mouse. Treatment began when the largest tumor reached approximately 100 mm^3^. According to the size of the tumor, the mice were allocated unbiasedly into four groups (eight mice per group): A, control; B, MLN8237 (5 mg/kg); C, MLN8237 (15 mg/kg); and MLN8237 (30 mg/kg). Drug or vehicle control was administered by gavage once daily for 18 days. The tumor size was measured every other day using an electronic caliper. Tumor volumes were calculated by the following formula: A × B^2^/2, where A is the greatest diameter, and B is the diameter perpendicular to A. Follow-up of individual mice was conducted. The animals were then euthanized, and tumor xenografts were immediately removed, weighed, stored, and fixed.

### Immunohistochemistry (IHC)

Tumors from the mouse xenograft model were dissected, fixed, and paraffin-embedded. Paraffin-embedded tumors were cut into 4-μm-thick slices. Tumor sections were dewaxed and rehydrated, endogenous peroxidase activity and nonspecific binding sites were blocked, and immediately following the antigen retrieval. Thereafter, the sections were stained with anti-Histone3 dimethyl (Arigo; ARG54763; 1:1000), anti-pHistone 3 (ser10) (Arigo; ARG51679; 1:1000). The secondary peroxidase-conjugated anti-rabbit antibodies were incubated and revealed with diaminobenzidine (DAB), followed by counterstaining with hematoxylin and image acquisition with a microscope (Olympus, Tokyo, Japan) at a magnification of 400×.

### Immunofluorescence

Cells were seeded in 8-well chamber slides (3 × 10^4^ cells/well) treated with MLN8237 or transfected with AURKA siRNA and then were washed with PBS and fixed with 50% methyl alcohol and 50% acetone at 4 °C for 30 min. Next, the cells were blocked with 1% BSA for 1 h at 37 °C. Thereafter, the cells were incubated with the primary AURKA antibody (abcam; ab52973; 1:500) diluted with blocking buffer at 4 °C overnight. Next, the cells were washed with PBS three times and incubated with the second antibody (abcam; ab150075; 1:200) for 1 h at 37 °C. The blue-fluorescent DAPI nucleic acid stain (Invitrogen; D1306) was used as a counterstain. The slides were mounted with coverslips, and the cells were visualized with a fluorescence microscope.

### MACS separation and tumor sphere formation

Neuroblastoma stem cells were separated with CD133^+^ microbeads (Miltenyi Biotec; 130-100-857) following the manufacturer’s instructions. For tumor sphere formation, the cells were seeded at a density of 1 cell/μl and were cultured in Dulbecco’s modified Eagle’s medium/Ham’s F-12 (1:1) containing 20 ng/ml of EGF (Gibco; PHG0315), 20 ng/ml of bFGF (R&D Systems; 233-FB-025), 1% l-glutamine, 1× Glutamax (Gibco; 12860-01) and 1× N21-MAX media supplement (R&D Systems; AR008).

### Statistical analysis

The data are representative of three independent experiments. The data were presented as the mean ± SD. Statistical analysis was performed using Prism 6 (GraphPad Software, Inc.) and SPSS v. 12.0 (SPSS, Inc.). The unpaired two-tailed Student’s t test was used to perform statistical comparison between two groups. ANOVA was used for multiple comparisons. P < 0.05 was considered to indicate statistical significance.

## Results

### MLN8237 induces cellular senescence, G2/M cell cycle arrest, and cell growth inhibition in the neuroblastoma cell line IMR32 in vitro

**Fig. 1:**
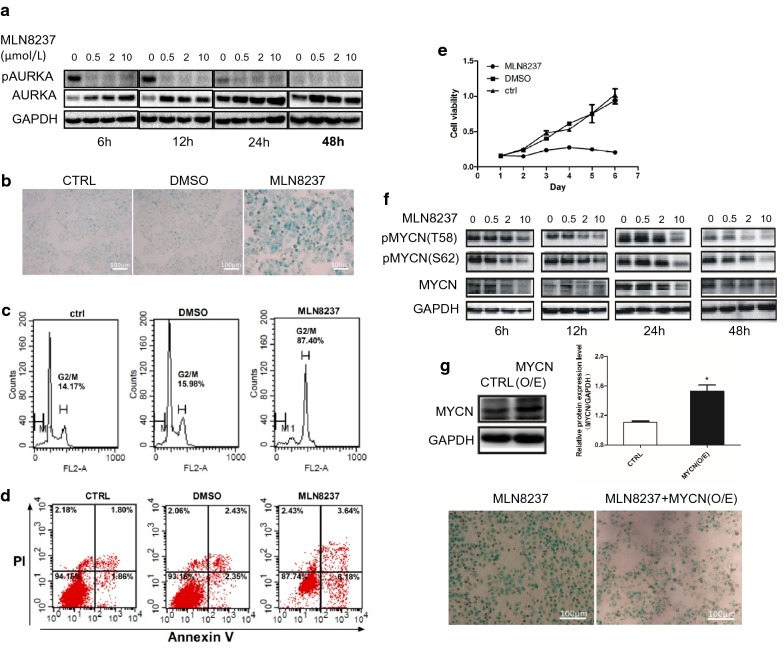
MLN8237 induced cell senescence, G_2_/M arrest,cell growth inhibition, and MYCN degradation in neuroblastoma cell line IMR32. **a**, **f** IMR32 cells were treated with 0, 0.5 μmol/l, 2 μmol/l and 10 μmol/l of MLN8237, respectively. Cell samples were collected at 6 h, 12 h, 24 h,and 48 h time points after MLN8237 treatment. Total proteins were extracted for western blot analysis for AURKA, pAURKA, MYCN, pMYCN(T58), and pMYCN(S62). **b** IMR32 cells were treated with 2 μmol/l of MLN8237, DMSO or no treatment as control. At day 3, cellular senescence was evaluated by SA-β-gal staining. Senescent cells show blue staining. Cell cycle **c** and apoptosis **d** analysis were assessed by flow cytometry. **e** Cell viability was assessed by MTT assay from day 1 to day 6 after 2 μmol/l of MLN8237 treatment, DMSO or no treatment as control. Each sample was analyzed by triplicates. Error bars correspond to the averages ± S.D. **g** IMR32 cells were treated with 2 μmol/l of MLN8237. Twenty-four hours later, cells were transfected with MYCN expression plasmid. Western blotting was used to verify MYCN expression. (upper figure). At 48 h after transfection, cellular senescence was evaluated by SA-β-gal staining (lower figure)

As an AURKA-targeting inhibitor, MLN8237 caused a pronounced decrease in phosphorylated AURKA (pThr288) at different time points in the neuroblastoma cell line IMR32 (Fig. 1a). β-Galactosidase (SA-β-gal) staining results showed that cellular senescence occurred at 24 h and peaked at 72 h after MLN8237 treatment. An enlarged and flattened morphology with an increased activity of SA-β-gal is the main feature of senescent cells (Fig. 1b). Cell cycle analysis revealed a significant increase in the number of cells at the G2/M phase (Fig. 1c). Flow cytometry showed that only a small number of cells undergo apoptosis (Fig. 1d), indicating that, under the stimulation of MLN8237, IMR32 cells mainly senesce but do not undergo apoptosis. The MTT assay showed that the inactivation of AURKA leads to a significant inhibitory effect on the growth of IMR32 cells (Fig. 1e). Taken together, these results provide evidence that IMR32 neuroblastoma cells undergo cellular senescence and growth inhibition due to AURKA inhibition.

### Inactivation of AURKA results in MYCN destabilization

Because the stabilization of MYCN is a critical function of AURKA in human neuroblastoma [7], we want to validate whether the inhibition of AURKA induces MYCN degradation in our study. As shown in Fig. 1f, both total MYCN and phosphorylated MYCN (T58 and S62) were decreased in a time- and dose-dependent manner after MLN8237 treatment. Next, we tested whether cell senescence is triggered by MYCN degradation. A rescue experiment of MYCN eukaryotic expression plasmid transfection into MLN8237-treated IMR32 cells was performed. The result of SA-β-gal staining indicated that exogenous MYCN expression weakened the occurrence of senescence (Fig. 1g), indicating that MYCN degradation resulting from AURKA inhibition is a possible cause of neuroblastoma cellular senescence.

### The cellular senescence-inducing p53/p21 pathway is involved in MLN8237-treated IMR32 neuroblastoma cells

**Fig. 2:**
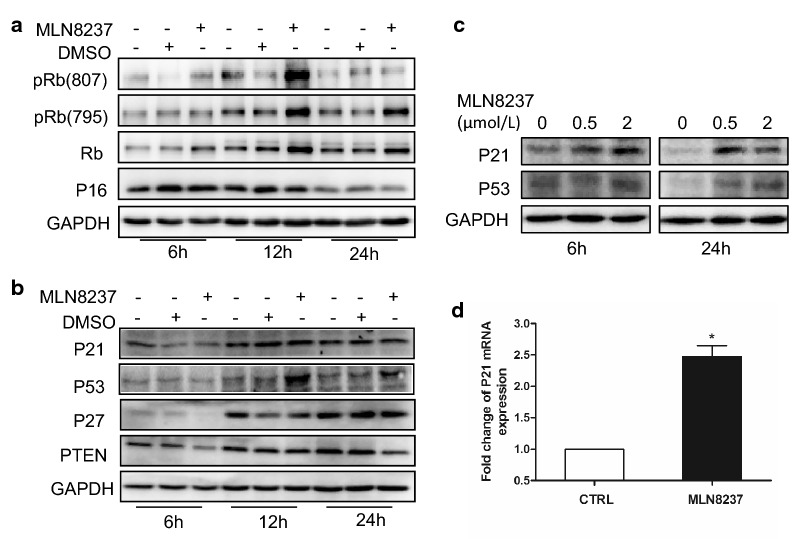
MLN8237 stimulated cellular senescence was mediated by P53 pathway. Senescence-associated signal pathways were detected by western blot. IMR32 cells were treated with 2 μmol/l of MLN8237. DMSO or no treatment as control. Cell samples were collected at 6 h, 12 h, and 24 h. Total proteins were extracted for western blot analysis for (**a**) p16, Rb, pRb (807), and pRb (796); **b** P27, PTEN, p53, and p21. **c** IMR32 cells were treated with 0, 0.5, and 2 μmol/l of MLN8237, respectively. At 6 h and 24 h, cells were collected, and total proteins were extracted for western blot analysis for p53 and p21. **d** At 48 h after MLN8237 treatment, IMR32 cells were collected and total RNA were extracted. P21 mRNA were quantified by real-time RT-PCR

We next asked which signaling pathways were involved in the cellular senescence induced by MLN8237. Cell cycle-dependent kinase inhibitors p16, p21, and p27 are regarded as key effectors of cellular senescence [11]. In our study, we tested three senescence-inducing pathways involving these inhibitors, namely, the p16/Rb pathway, PTEN/p27 pathway, and p53/p21 pathway. p16, also known as p16^*INK4a*^, plays a role in inhibiting cyclin-dependent kinase 4 (CDK4) to slow down the cell cycle from G1 phase to S phase [12]. The retinoblastoma tumor suppressor protein (Rb) is the main substrate of CDK4/6, which drive cells through G1 into S phase. CDK4/6 binds cyclinD and forms an active protein complex that phosphorylates Rb. Once phosphorylated, pRB dissociates from the transcription factor E2F1. This liberates E2F1 from its bound state in the cytoplasm and allows it to enter the nucleus to promote the transcription of target genes that are essential for transition from G1 to S phase [13]. In this study, the level of p16 showed no significant change in response to MLN8237 treatment, as shown in Fig. 2a, whereas both Rb and pRb were upregulated after MLN8237 stimulation. This indicates that the phosphorylation of Rb in MLN8237-stimulated cells does not occur via p16 and that cell senescence induced by MLN8237 is not mediated by the p16/Rb pathway.

Tumor suppressor PTEN is a phosphatase that catalyzes the conversion of 3,4,5-trisphosphate (PIP3) into PIP2, thus opposing the actions of PI3K/Akt [14]. It was found that overexpression of PTEN results in the upregulation of p27, which is a cyclin-dependent kinase inhibitor 1B (p27^Kip1^), binds to and prevents the activation of cyclin E-CDK2 or cyclin D-CDK4 complexes, thus controlling the cell cycle progression at G1 [15]. In IMR32 cells, both PTEN and p27 were downregulated after MLN8237 treatment, as shown in Fig. 2b. It seems certain that the PTEN/p27 pathway plays no role in MLN8237-induced cell senescence.

p21 (alternatively p21^Cip1^), also known as cyclin-dependent kinase inhibitor 1 or CDK-interacting protein 1, is a cyclin-dependent kinase inhibitor (CKI) that can inhibit all cyclin/CDK complexes [16]. p21 represents a major target of p53 activity and, thus, is associated with linking DNA damage to cell cycle arrest [17]. In our study, the level of p53 was markedly upregulated, especially at 12 h after MLN8237 treatment (Fig. 2b). Next, we tested the protein level of p53 and p21 at different time points with different doses of MLN8237. The results showed that both p53 and p21 were upregulated (Fig. 2c). The mRNA of p21 was increased as well (Fig. 2d). Altogether, MLN8237-induced IMR32 cellular senescence is mediated by the p53/p21 pathway.

### MLN8237 induces tumor growth inhibition in a xenograft mouse model

**Fig. 3:**
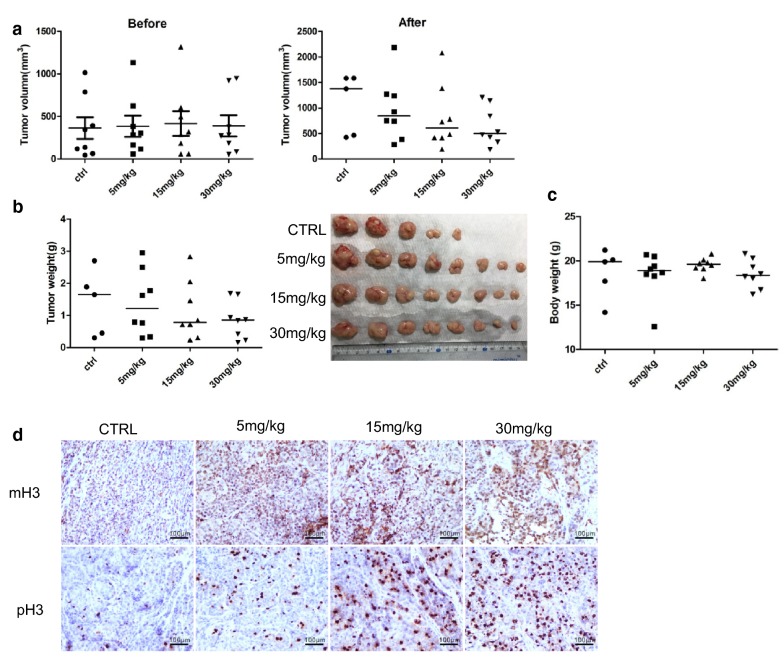
MLN8237 inhibited tumor growth in mice model. A total of 5 × 10^6^ of IMR32 cells were subcutaneously inoculated in the right flank of each BALB/c nude mice. When the largest tumor reached about 100 mm^3^, the mice were randomly divided into four groups and given MLN8237 (30 mg/kg, 15 mg/kg, 5 mg/kg, and 0 mg/kg, respectively) once daily by oral gavage. **a**–**c** When tumors reached a size of 1.8 cm diameter, mice were sacrificed. Tumor volume (before and after treatment), tumor weight, and mice body weight were analyzed among the different group. **d** IHC staining was performed to analyse phospholyrated H3 and methylated H3K9

We further examined the in vivo antitumor effect of MLN8237 using the nude mouse xenograft model. Before drug treatment, tumor-bearing mice were divided equally into groups according to the size of tumors. At the end of the experiment, the growth of tumors in drug-treated mice was significantly inhibited (Fig. 3a, b). The median tumor size was 499.77 mm^3^ in 30 mg/kg of the MLN8237-treated group, 605.61 mm^3^ in 15 mg/kg of the MLN8237-treated group, 841.78 mm^3^ in the 5 mg/kg of the MLN8237-treated group, and 1375.4 mm^3^ in the control group. The median tumor weight of the MLN8237-treated groups was lower than that of the vehicle-treated group (30 mg/kg: 855 mg; 15 mg/kg: 785 mg; 5 mg/kg: 1210 mg; Ctrl: 1650 mg). The median body weight of mice showed no significant difference between the MLN8237-treated and control group (30 mg/kg: 18.36 g; 15 mg/kg: 18.95 g; 5 mg/kg: 18.59 g; Ctrl: 19 g) (Fig. 3c). Both the phosphorylated and methylated H3 were upregulated in MLN8237-treated mice, demonstrating that MLN8237 also induced G2/M cell cycle arrest and cell senescence in vivo (Fig. 3d). Together, the results demonstrated a certain dose of MLN8237 can inhibit the growth of transplanted tumors in mice.

### MLN8237 treatment results in unexpected activation of Akt/Stat3

**Fig. 4:**
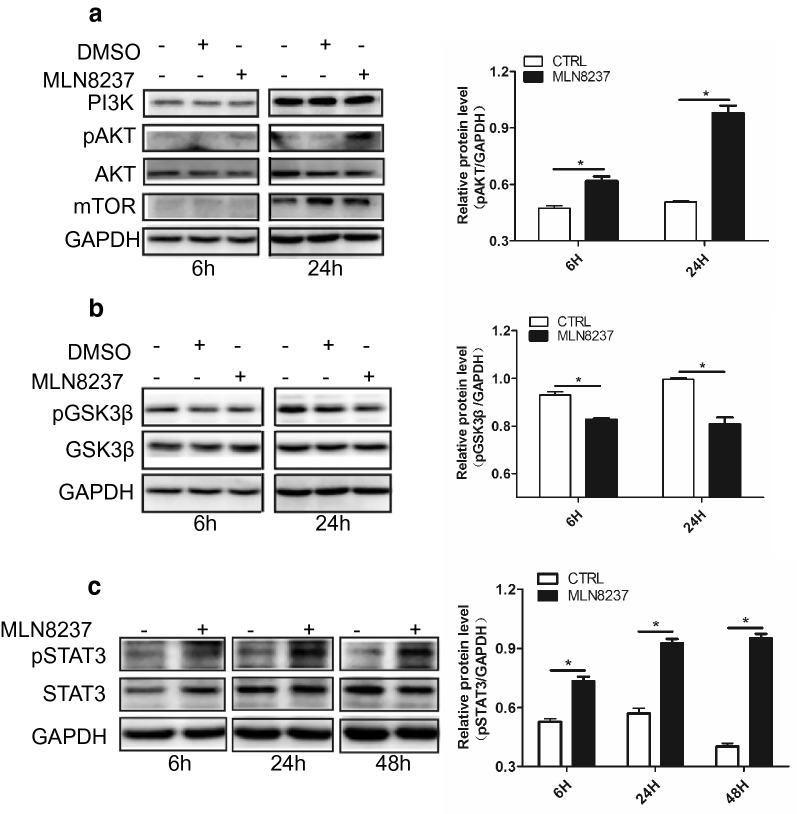
MLN8237 treatment resulted in abnormal activation of Akt/Stat3 pathway. IMR32 cells were treated with 2 μmol/l of MLN8237. DMSO or no treatment as control. Cell samples were collected at 6 h and 24 h. Total proteins were extracted for western blot analysis for **a** Akt, pAkt, and mTOR; **b** GSK3β and pGSK3β; **c** Stat3 and pStat3. Gray values were calculated by imageJ software. The data were shown as the mean ± SEM of three independent experiments. *P < 0.05

PTEN is known to control DNA repair, cell proliferation and survival, guards the genome against structural and numerical chromosome instability and is an essential tumor suppressor gene that encodes a phosphatase protein that antagonizes the PI3K/AKT/mTOR antiapoptotic pathway. Because MLN8237 can induce PTEN downregulation, we speculated whether MLN8237 activated Akt. As expected, Akt was activated in response to MLN8237 stimulation (Fig. 4a). Activated AKT not only can transduce antiapoptotic signals by phosphorylating and inactivating key proteins involved in cell proliferation and survival, making it difficult to control tumor progression, but also affects cell proliferation via modulation of the cell cycle machinery and regulation of the activity of the cyclin D1 kinase glycogen synthase kinase-3β(GSK-3β) [18]. We found MLN8237 treatment induced pGSK3β downregulation (Fig. 4b). However, as mentioned in the literature, the MYC family transcription factors are destabilized by phosphorylation of GSK-3β; thus, the downregulated pGSK-3β can subsequently prevent the degradation of MYCN protein, representing another drawback for NB treatment.

pAkt can activate signal transducer and activator of transcription 3 (STAT3) through the Akt/Stat3 pathway [19]. In our study, phosphorylated Stat3 (pStat3) was significantly upregulated (Fig. 4c). Stat3 is constitutively activated in many cancers and plays a pivotal role in tumor growth and metastasis. It can regulate cellular proliferation, migration, invasion, and angiogenesis. Persistent activation of Stat3 can function as a master regulator of molecular and biological events and promotes growth and inhibits apoptosis. Hence, although MLN8237 induced IMR32 cells into a senescence state, there is still a risk of cells surviving and re-entering the cell cycle.

### “Stemness” increases in senescent cells induced by MLN8237 treatment

**Fig. 5:**
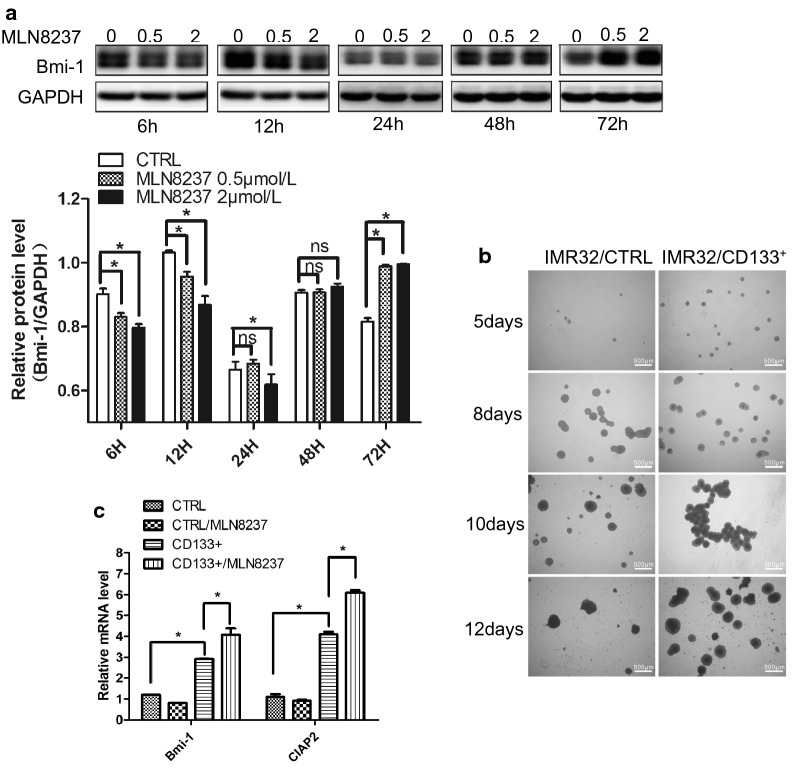
MLN8237 treatment induced IMR32 cell “stemness” enhancement. **a** IMR32 cells were treated with 0, 0.5 μmol/l, and 2 μmol/l of MLN8237, respectively. Cell samples were collected at 6 h, 12 h, 24 h, 48 h, and 72 h time points. Total proteins were extracted for western blot analysis for Bmi1 protein expression. Gray values were calculated by imageJ software. The data were shown as the mean ± SEM of three independent experiments. *P < 0.05. **b** CD133^+^ cells were separated from IMR32 by using CD133 MicroBead Kit (Miltenyi Biotec). CD133^+^ cells and CD133^−^ cells were seed in ultra-low adhesion culture dishes to culture for 12 days, respectively. At 5 days, 8 days, 10 days, and 12 days time points, microsphere formation were observed by microscope photography. **c** At 12 days, cell microsphere were collected and digested to seed into 6-well plate. MLN8237 treated for 48 h and total RNA were extracted. The mRNA levels of Bmi1 and CIAP2 were determined by real time RT-PCR. The data were shown as the mean ± SEM of three independent experiments. *P < 0.05

After treatment with MLN8237, IMR32 cells survived for at least two weeks and then died. Given that many growth factors support cell growth in the tumor microenvironment in vivo, we speculate that the fate of MLN8237-induced senescent cells is not death but likely re-entry into the cell cycle. This phenomenon may explain why MLN8237 does not work well in clinical trials. In cell culture systems, because of the lack of cytokine support, senescent cells cultured in normal medium eventually die. However, when normal medium is replaced with neural stem cell culture medium, the senescent cells resume division and proliferation (our unpublished result). B-lymphoma MMLV insertion region 1 (Bmi1) is a stem cell marker expressed in 90% of primary NB and plays a crucial role in the pathogenesis of NB [20]. We detected the level of Bmi1 in IMR32 after MLN8237 treatment. The expression of Bmi1 was inhibited at the initial stage from 6 h to 24 h in the presence of MLN8237. However, with the prolongation of treatment, at 72 h, Bmi1 was upregulated (Fig. 5a). To observe the effect of MLN8237 on tumor stem cells, we used CD133 magnetic beads to isolate tumor stem cells in IMR32 cells. The isolated CD133^+^ cells were tested for microsphere formation, as shown in Fig. 5b. CD133^+^ cell microspheres were collected, digested by trypsin and seeded into six-well plates. At 48 h after MLN8237 treatment, the mRNA expression levels of Bmi1 and CIAP2 were detected by quantitative RT-PCR. The results showed that, after MLN8237 treatment, the expression levels of Bmi1 and CIAP2 in IMR32/CD133^+^ cells were significantly higher than those in IMR32/CTRL cells (Fig. 5c). This means MLN8237 stimulates the cell stemness and antiapoptosis of IMR32 cells.

### MLN8237 leads to abnormal high expression of AURKA protein in vitro and in vivo

**Fig. 6:**
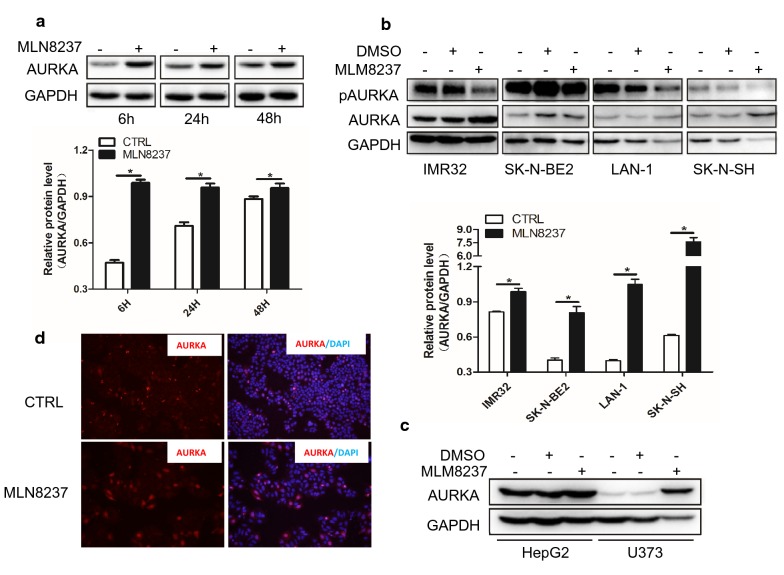
MLN8237 treatment led to abnormal high expression of AURKA in vitro. **a** IMR32 cells were treated with 2 μmol/l of MLN8237. Cell samples were collected at 6 h, 24 h, and 48 h time points. Total proteins were extracted for western blot analysis for AURKA protein expression. **b** Different neuroblastoma cell lines including IMR32, SK-N-BE2, LAN-1, and SK-N-SH were treated with 2 μmol/l of MLN8237. At 48 h, total proteins were extracted for western blot to test AURKA and pAURKA expression. Gray values were calculated by imageJ software. The data were shown as the mean ± SEM of three independent experiments. *P < 0.05. **c** Hepatocellular carcinoma cell line HepG2 and glioma cell line U373 were treated with 2 μmol/l of MLN8237. At 48 h, total proteins were extracted for western blot to test AURKA and pAURKA expression. **d** 1.0 × 10^5^ of IMR32 cells were seed into 8-well chamber slide. The next day, cells were treated with 2 μmol/l of MLN8237. At forty-eight hours after transfection, cells were fixed and Immunofluorescence staining was performed to test AURKA expression

Although MLN8237 inhibits the activity of AURKA, we unexpectedly found that the protein level of AURKA was upregulated in IMR32 cells after MLN8237 treatment, as shown in the western blot results (Figs. 1a, 6a). To verify this result, we tested three other neuroblastoma cell lines—SK-N-BE2, LAN-1, and SK-N-SH. Without exception, AURKA levels in all four cell lines were upregulated to varying degrees after MLN8237 treatment (Fig. 6b). We also investigated AURKA protein level in non-neuroblastoma cell lines including HepG2 and U373 upon MLN8237 treatment. The same results were shown in Fig. 6c. Immunofluorescence results showed that MLN82137 treatment not only increased the protein level of AURKA but also changed the distribution of AURKA, from centrosome location into diffuse distribution in both the cytoplasm and nucleus (Fig. 6d).

**Fig. 7:**
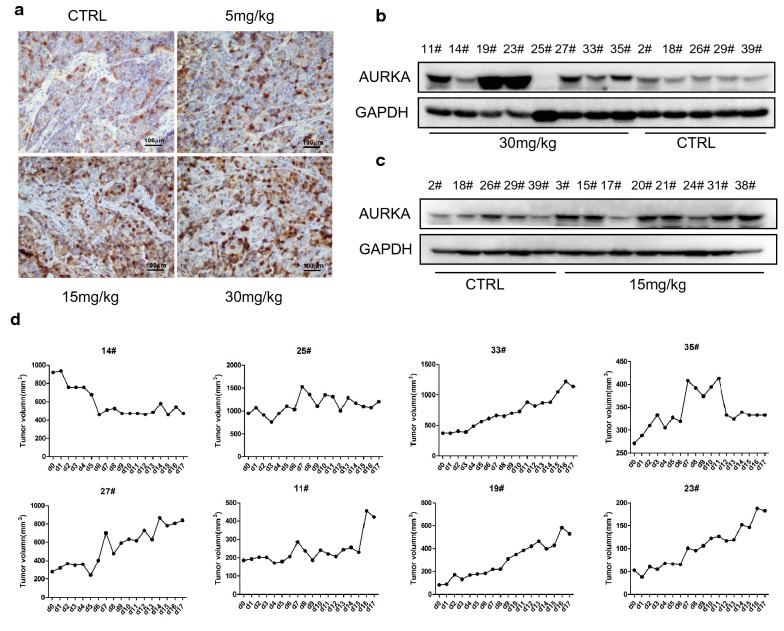
MLN8237 treatment led to abnormal high expression of AURKA in vivo. **a** At the end of the treatment, the tumor tissues were removed from the transplanted tumor model of mice and made into paraffin sections. The tissue slides derived from mice model treated with 5 mg/kg of MLN8237, 15 mg/kg of MLN8237, 30 mg/kg of MLN8237, respectively. IHC staining was performed to analyse AURKA expression. **b** Extraction of total protein from tumor tissues and detection of AURKA expression by western blot. The level of total AURKA was compared between 30 mg/kg treatment group, 15 mg/kg treatment group **c**, and control group, respectively. **d** The tumor sizes of each mouse in 30 mg/kg of MLN8237 group were monitored during the period of treatment

Next, we checked the level of AURKA in the tumor tissue from xenograft mice. IHC results showed that the AURKA levels were significantly increased in the MLN8237 treatment groups, including the 5-mg/kg group, 15-mg/kg group, and 30-mg/kg group (Fig. 7a). Additionally, the level of AURKA was detected in each mouse of the 30-mg/kg group and 15-mg/kg group (Fig. 7b, c). The tumor size of mouse 14# in the 30-mg/kg group was significantly smaller at the treatment ending point. In mouse 25#, the tumor size was not enlarged under MLN8237 treatment (Fig. 7d). Thus, in the two mice, 14# and 25#, the levels of AURKA were not affected by MLN8237 treatment. These results showed that the expression level of AURKA in each mouse in MLN8237-treated group was associated with the tumor size (Fig. 7b, d), indicating that MLN8237-induced AURKA upregulation may lead to a less than expected effect of MLN8237 on neuroblastoma.

### Knockdown of AURKA induces cell apoptosis and reduces cell survival via the inhibiting Akt/Stat3 pathway

**Fig. 8:**
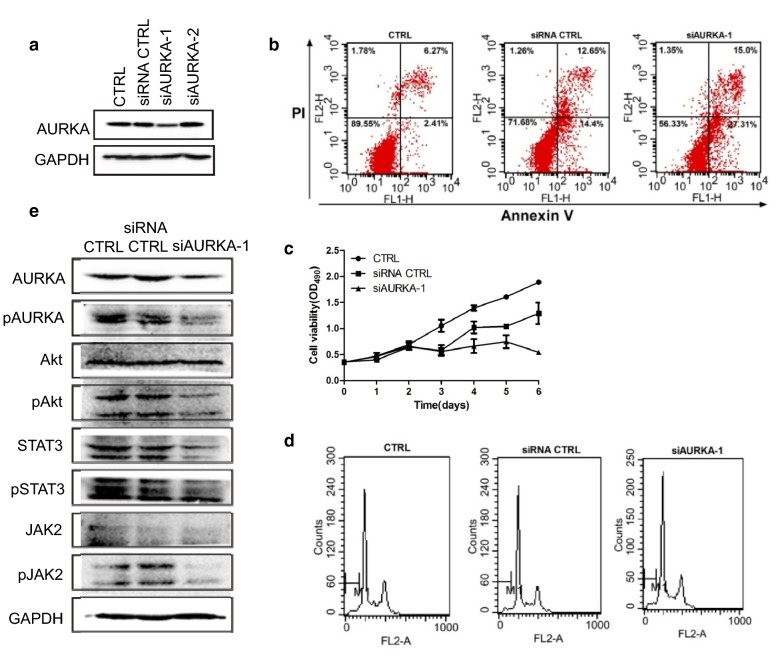
AURKA knockdown induced cell apoptosis and cell growth inhibition by repressing the activity of Akt/Stat3 pathway (**a**) 2.0 × 10^5^ of IMR32 cells were seed into 6-well plate. The next day, cells were transfected with AURKA siRNAs, siAURKA-1, si-AURKA-2, or siRNA CTRL. At 48 h after transfection, cells were harvested and total protein were extracted. Western blotting was performed for AURKA expression. **b** 2.0 × 10^5^ of IMR32 cells were seed into 6-well plate. The next day, cells were transfected with AURKA siRNA-1. At 48 h after transfection, cells were harvested and cell apoptosis and cell cycle analysis (**c**) were was performed by flow cytometry. **d** 5 × 10^3^ of IMR32 cells were seed into 96-well plate. The next day, cells were transfected with AURKA siRNA-1. At d0, d1,d2,d3,d4,d5,d6 time points, MTT assay was adopted for cell vialility test. Each sample was analyzed by triplicates. Error bars correspond to the averages ± S.D. **e** 2.0 × 10^5^ of IMR32 cells were seed into 6-well plate. The next day, cells were transfected with AURKA siRNA-1. At 48 h after transfection, cells were harvested and western blot was performed for expression of AURKA, pAURKA, Akt, p-Akt, STAT3, p-STAT3, JAK2, and p-JAK2

Because of the abnormal high expression of AURKA in MLN8237-treated neuroblastoma cells, knockdown of AURKA should have an inhibitory effect on cell growth. Two small interfering RNAs (siRNAs) of AURKA were synthesized and transfected into IMR32 cells. SiAURKA-1 was selected to perform subsequent experiments according to the interfering effect test (Fig. 8a). The results showed that AURKA knockdown induced cell apoptosis and inhibited cell growth (Fig. 8b, c). No effect of AURKA siRNA on cell cycle arrest was observed (Fig. 8d). SA-β-gal staining showed that no senescent cells appeared after AURKA siRNA transfection (data not shown). Because AURKA can activate the phosphorylation and transcription activities of Stat3, we evaluated the Jak2/Stat3 pathway and found that knockdown of AURKA decreased the activity and protein levels of Jak2 and Stat3 (Fig. 8e). Although the protein level of Akt was not affected by AURKA siRNA, phosphorylated Akt was decreased significantly (Fig. 8e). Taken together, AURKA knockdown induces cell apoptosis and reduces cell survival via inhibiting the Jak2/Stat3 pathway and deactivating Akt.

### Downregulation of AURKA forces senescent cell into a state of cell death via deactivating Akt/Stat3

**Fig. 9:**
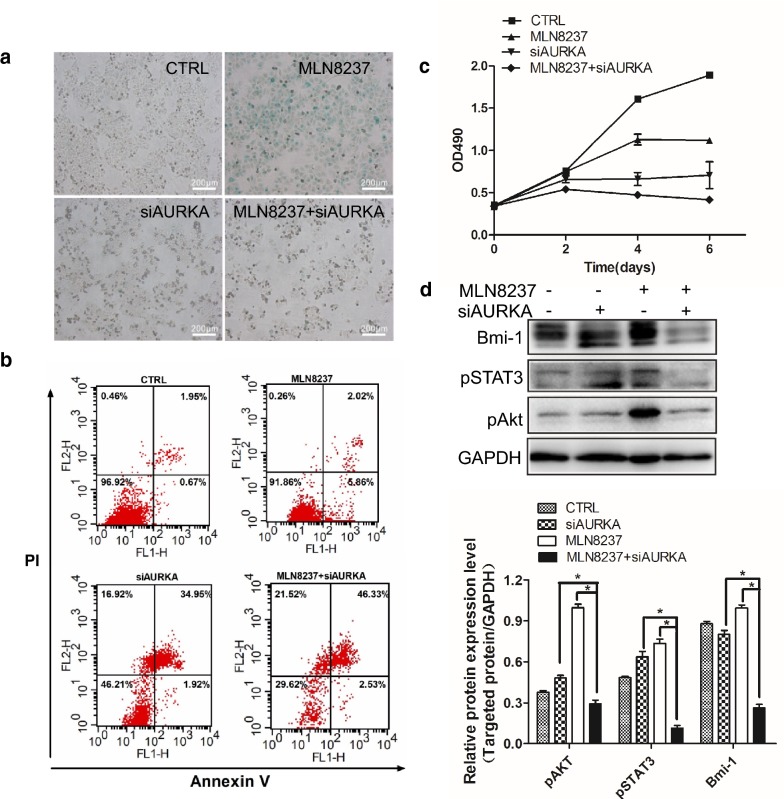
MLN8237 treatment followed by knockdown of AURKA forced senescent cells into apoptosis. **a** IMR32 cells were treated with 2 μmol/l of MLN8237. Two hours later, MLN8237 withdrawal followed by siAURKA-1 transfection. At 24-h after transfection, removing the supernatant, cells were cultured in normal medium in the presence of 2 μmol/l of MLN8237 till 72 h. Cellular senescence was evaluated by SA-β-gal staining, cell apoptosis (**b**) was assessed by flow cytometry. Cell viability (**c**) was assayed by MTT method from day 1 to day 6 after 2 μmol/l of MLN8237 treatment, or siAURKA transfection alone, or MLN8237 treatment plus siAURKA transfection, or no treatment as control. Each sample was analyzed by triplicates. Error bars correspond to the averages ± S.D. **d** IMR32 cells were treated with 2 μmol/l of MLN8237. At 48 h after transfection, cells were harvested and total protein were isolated. Western blots were assayed for pAkt, pSTAT3, and Bmi1. Gray values were calculated by imageJ software. The data were shown as the mean ± SEM of three independent experiments. *P < 0.05

Because MLN8237 can upregulate AURKA protein expression, we wondered whether AURKA siRNA could be utilized to decrease the AURKA level after MLN treatment. From Fig. 9a, blue-staining globular cells were significantly decreased in the presence of MLN8237 treatment followed by siAURKA transfection, indicating that MLN8237-induced senescent cells were forced to undergo apoptosis in response to AURKA knockdown, as shown in Fig. 9b. Cell viability assay validated that MLN8237 treatment plus AURKA siRNA transfection inhibited cell growth more than any single treatment (Fig. 9c). We also tested the levels of Bmi1, pStat3, and pAkt and found that all were inhibited by the combination treatment of MLN8237 and siAURKA.

## Discussion

In human neuroblastoma, amplification of the *MYCN* gene predicts a poor prognosis and resistance to therapy. Because of a lack of a “druggable” binding pocket, MYCN protein was challenging as a direct antitumor target. Aurora kinase A (AURKA) is an oncogenic serine/threonine kinase that can cause cell transformation and centrosome amplification when overexpressed. Moreover, in neuroblastoma, stabilization of MYCN is a critical function of AURKA. MYCN forms a complex with AURKA that protects MYCN from proteasomal degradation. While considered to be indirectly “druggable” therapeutically, MYCN can be destabilized posttranscriptionally by the inhibition of AURKA. As an AURKA inhibitor, MLN8237 at least plays two roles. On the one hand, MLN8237 abolished the autophosphorylation of AURKA at Thr288 (T288) and caused cells to fail to divide properly, caused cell cycle arrest at G2/M phase, and cellular senescence appeared. On the other hand, via dissociation of an AURKA/MYCN complex, MLN8237 treatment induced a time- and dose-dependent decrease in the MYCN protein level, independent of its kinase activity. MYCN is released from the AURKA/MYCN complex because of MLN8237 disruption. Ubiquitin ligase Fbxw7 can then bind to MYCN and promote its degradation. Fbxw7 recognizes MYCN after sequential phosphorylation at S62 by cyclin B/CDK1, which primes MYCN for phosphorylation at T58 by GSK3β [21]. We also speculate that the slow kinetics of the decrease in total MYCN in our study is probably due to the requirement for mitotic phosphorylation of MYCN. In our study, the rescue of MYCN expression can reduce the occurrence of senescence induced by MLN8237 treatment, indicating that lowering the MYCN level may contribute to cellular senescence.

MLN8237 treatment induced neuroblastoma cell line IMR32 senescence in vitro. The proportion of senescent cells is almost up to 100% after MLN8237 treatment. In this case, IMR32 cells are an excellent model to study therapeutic-induced cell senescence. We detected the MLN8237-induced senescence-related signal pathways, including the p53/p21, p16/Rb, and PTEN/p27 pathways. MLN8237 induced the upregulation of p53 and p21. Although p16 was upregulated in cell replicative senescence [22], there was no significant change in MLN8237-induced senescence. Phosphorylation of Rb was elevated, indicating the inactivation state of Rb in the presence of MLN8237. Moreover, both PTEN and p27 were downregulated in response to MLN8237 stimulation. As a negative regulator of PI3K/AKT, the downregulation of PTEN was accompanied by the upregulation of AKT activity. From our results, Akt was activated after MLN8237 treatment, followed by attenuation of pGSK3β activity. This directly led to the downregulation of MYCN phosphorylation and hindrance of MYCN degradation. Additionally, we observed a significant increase in phosphorylated Stat3. Given the role of the Stat3 pathway in cancer development, this news is disappointing for anticancer treatment.

Although MLN8237 induces IMR32 cell senescence, senescent cells are viable and survive, even though they have active DNA damage responses. Indeed, senescent cells are better able to withstand stresses such as serum deprivation than nonsenescent cells [23]. In our study, we observed that, following the drug withdrawal of MLN8237 after 3 days of treatment, IMR32 cells still survived for several weeks in normal medium. If the normal medium is replaced with a stem cell culture medium, the cells re-enter the cell cycle. We isolated tumor stem cells from IMR32 cells by CD133^+^ magnetic beads and observed their response to MLN8237. The results showed that, after MLN treatment, the expression levels of Bmi1 and CIAP2 in CD133^+^ IMR32 cells were significantly higher than those in total IMR32 cells. Thus, if the senescent cells induced by MLN8237 treatment in vivo are not cleared as soon as possible, the tumor is likely to relapse. From the literature, in vivo senescent cells appeared to be removed by the immune system, rather than apoptosis or necrosis [24]. Together with our results, we hypothesized that antiapoptotic, pro-survival mechanisms could be upregulated in senescent cells, and interfering with these protective mechanisms might achieve selective elimination of senescent cells.

We tried to use an Akt inhibitor or a Stat3 inhibitor in combination with MLN8237, but the effect was negligible (data not published). Our focus returned to AURKA itself because we surprisingly found that the AURKA protein level rose in cells after MLN8237 treatment. Additionally, this is not the isolated case. In all cell lines we tested, including SK-N-BE(2), SK-N-SH, and LAN-1, this phenomenon exists. We also used non-neuroblastoma cell lines, the hepatocarcinoma cell line HepG2 and glioma cell line U373. AURKA was upregulated in both cell lines, without exception. In the mouse model, the upregulation of AURKA was positively correlated with the size of the transplanted tumor in the presence of MLN8237 treatment. AURKA is carcinogen, and its high expression is associated with a poor prognosis. From our results, MLN8237 treatment induced not only AURKA overexpression but also its abnormal distribution. Unlike in normal IMR32 cells, AURKA is located at the centromere. In senescent IMR32 cells, AURKA is distributed in both the nucleus and cytoplasm. The abnormal distribution of AURKA leads to chromosomal instability, and cells lose the ability to divide properly. More importantly, AURKA has a transactivating function independent of its kinase activity. For example, using a luciferase reporter assay, Ahmed Katsha et al. demonstrated that AURKA expression induced the transcriptional activity of Stat3 [25]. AURKA regulates survivin stability through targeting FBXL7, and AURKA regulated FBXL7 both at the levels of transcription and translation [26]. Nuclear AURKA acts as a transcriptional factor that activates the *MYC* promoter to enhance breast cancer stem cell phenotype independent of its kinase activity [27]. In our study, knockdown of AURKA by RNAi induced approximately 50% of IMR32 cell apoptosis. The activities of the Jak2/Stat3 and Akt pathways were inhibited. From this point, MLN8237 combined with AURKA siRNA has a superposition effect on neuroblastoma cells. As expected, MLN8237 combined with AURKA siRNA significantly inhibited IMR32 cell growth. MLN8237-induced senescence cells undergo apoptosis in the presence of AURKA siRNA. Both pAkt and pStat3 are inhibited, indicating that the downregulation of AURKA in senescent cells forced them into a state of cell death via deactivating cell surviving pathways.

In summary, as an AURKA inhibitor, MLN8237 can induce neuroblastoma cell senescence, G2/M cell cycle arrest, and cell growth in vitro and in vivo. Moreover, MLN8237 upregulates inhibition the AURKA protein level, activates the Akt/Stat3 pathway, and increases the antiapoptosis activity of tumor-initiating cells. AURKA knockdown decreases the Akt/Stat3 activity and induces cell apoptosis. The combination of MLN8237 with AURKA siRNA can effectively control NB cell growth via cell senescence induced by MLN8237 and subsequent removal of senescent cells by AURKA siRNA-induced apoptosis. Thus, the effect of AURKA-targeted inhibition of tumor growth plays roles in both the deactivation of AURKA activity and the decrease in the AURKA protein level.

## Conclusions

To improve the effects of AURKA targeting inhibition on neuroblastoma growth needs not only inactivation of AURKA, but also down-regulation of AURKA protein level. AURKA knockdown contributes to the eradication of senescent tumor cells induced by AURKA inhibitors. These findings could help researchers properly evaluate clinical trials of AURKA inhibitors and give hints in clinical drug development for neuroblastoma.

## Data Availability

The datasets during and/or analysed during the current study available from the corresponding author on reasonable request.

## Abbreviations

AURKA: aurora kinase A
NB: neuroblastoma
siRNA: small interfering RNA
SA-β-gal: senescence-associated β-gal
IHC: immunohistochemistry
Bmi1: B-lymphoma MMLV insertion region 1
Akt/Stat3: protein kinase B/signal transducer and activator of transcription 3
MTT: 3-(4,5-dimethylthiazol-2-yl)-2,5-diphenyltetrazolium bromide
